# Perceptions and attitudes towards Covid-19 vaccines: narratives from members of the UK public

**DOI:** 10.1007/s10389-022-01728-w

**Published:** 2022-06-30

**Authors:** Btihaj Ajana, Elena Engstler, Anas Ismail, Marina Kousta

**Affiliations:** 1 Department of Digital Humanities, King’s College London, Strand Campus, London, WC2R 2LS UK; 2 Department of Basic and Clinical Neuroscience, Maurice Wohl Clinical Neuroscience Institute, King’s College London, 5 Cutcombe Road, London, Brixton SE5 9RT UK; 3 Department of Population Health and Environmental Sciences, King’s College London, Guy’s Campus, London, SE1 1UL UK; 4 Department of Global Health & Social Medicine, King’s College London, Bush House, North East Wing, 40 Aldwych, London, WC2B 4BG UK

**Keywords:** Covid-19, Health, Pandemic, Public attitudes, Vaccines, Trust

## Abstract

**Aim:**

The aim of the paper is to enhance understanding of how members of the public make sense of the Covid-19 vaccines and to understand the factors influencing their attitudes towards such artefacts of pandemic governance.

**Methods:**

The paper draws on 23 online in-depth interviews with members of the UK public and builds on relevant literature to examine participants’ perceptions of the benefits and risks of Covid-19 vaccines, the sources that have shaped their attitudes, and the level of trust they have towards the government’s handling of the pandemic through vaccines.

**Results:**

The findings indicate that participants generally felt that the benefits of having the vaccine outweigh the risks and that Covid-19 vaccines are a crucial mechanism for enabling society to return to normal. Vaccine acceptance was, for some, strongly linked to a sense of social responsibility and the duty to protect others. However, some participants expressed concerns with regard to the side-effects of Covid-19 vaccines and their perceived potential impact on fertility and DNA makeup. Participants used various sources of information to learn about Covid-19 vaccines and understand their function, benefits, and risks. The majority of participants criticised the government’s response during the early stages of the pandemic yet felt positive about the vaccine rollout.

**Conclusion:**

Just as with any other vaccination programme, the success of the Covid-19 immunisation campaigns does not only depend on the efficacy of the vaccines themselves or the ability to secure access to them, but also on a myriad of other factors which include public compliance and trust in governments and health authorities. To support an effective immunisation campaign that is capable of bringing the pandemic to an end, governments need to understand public concerns, garner trust, and devise adequate strategies for engaging the public and building more resilient societies.

## Introduction

In their book *The Politics of Vaccination: A Global History*, Holmberg et al. (2017) remind us that government-led vaccination campaigns are political projects as much as they are health projects. Such campaigns seek to shape the immunity of entire populations in their attempt to contain and eliminate the spread of viruses. And as with many other governmental interventions and biopolitical forms of management, mass vaccination induces anxiety in some members of the public whilst arousing sentiments of solidarity and civic duty in others. As argued in the book’s introduction, ‘controversy clings to immunisation programmes,’ and different social groups have, at different times and places, disputed, evaded, or actively welcomed and demanded public health immunisation and the development of new vaccines (Holmberg et al. 2017: 1). Such arguments resonate strongly with the current context of Covid-19 vaccines, whose worldwide rollout has been subject to controversy as well as hope, indicating how immunisation campaigns are never neutral practices, but carry with them a host of values and presumptions about the role of governments, healthcare institutions, and private companies vis-à-vis public health, as well as the individual’s sense of obligation towards self and others.

The success of vaccination campaigns is seldom dependent only on the vaccines themselves. It depends also on a multitude of other factors including public compliance, vaccine acceptance, and trust in public health authorities and political leadership (Iftekhar et al. 2021). These factors are themselves influenced by various socio-cultural, political, and economic forces which, when intersecting with changing policy recommendations and constant media coverage, can have an impact on willingness to get vaccinated and on public responses to Covid-19 restrictions and pandemic management techniques. As such, it is important to gain an understanding of such factors and their interface with public opinions and attitudes. In this paper, we draw on a qualitative study we conducted from April to June 2021 on public perceptions and attitudes towards Covid-19 vaccines in the UK. The study involved 23 in-depth interviews with members of the UK public as well as a review of available literature on the subject. We asked participants about their understanding of vaccines’ benefits and risks, the sources from which they obtained their information and knowledge about the vaccines, and the levels of trust they had towards the government’s handling of the pandemic. Here, we report on the study’s findings and synthesise existing key literature by way of providing some background for the ensuing discussion.

## Background literature

The literature that has developed since 2020 on public perceptions of Covid-19 vaccines has been largely based on measuring and evaluating the public’s willingness to be vaccinated. A wide range of factors that could be related to a more positive (or negative) attitude towards these vaccines have been addressed across different countries and contexts. For instance, a quantitative study by Sallam et al. (2021) found that in East and South East Asia, acceptance rates among the general public are relatively high compared to other countries. Conversely, the Middle East and Eastern Europe have some of the lowest acceptance rates in the world (Sallam et al. 2021).

Covid-19 vaccines have raised a lot of controversy. Beliefs about these vaccines can affect the public’s willingness and compliance in getting vaccinated which, in turn, could evolve into a public health challenge considering the effect of the Covid-19 pandemic and the rapid spread of recent variants. Most common concerns relating to vaccine hesitancy are fears over potential side-effects, safety, and efficacy/benefits (Neumann-Böhme et al. 2020; Bell et al. 2020). There has been a recurring concern that these vaccines could be dangerous because of how quickly they were developed (Bell et al. 2020; Troiano and Nardi 2021; Biswas et al. 2021; Nguyen 2021). One study reported concerns that Covid-19 vaccines were potentially experimental, lacking enough studies regarding their side-effects, and that they might not be safe for certain subgroups, such as pregnant women and people with pre-existing conditions (Neumann-Böhme et al. 2020). Perceptions of the vaccines’ efficacy are consistently associated with vaccination intention, where higher efficacy is linked to increased Covid-19 vaccine acceptance (Malesza and Wittmann 2021). In some studies, individuals reported they were open to vaccination at a later time, after they see if the vaccine was safe for others (Nguyen 2021).

Conspiracy theories are a common factor associated with the Covid-19 vaccine uncertainty (Harambam 2020; Önnerfors 2021). As mentioned earlier, the Middle East is one of the regions with the lowest Covid-19 vaccine acceptance rates globally, which is assumed to be related to the dominance of conspiracy beliefs and a negative attitude towards vaccination (Sallam et al. 2021). In Jordan, Kuwait, and other Arab countries, beliefs that the purpose of Covid-19 vaccines is to inject microchips into recipients and induce infertility were more prevalent among females, lower educated individuals, and those who primarily used social media as a source of news and information (Sallam et al. 2021). A study from the United State also found that people who were not willing to take the vaccine were more likely to believe in vaccine myths and sceptical of scientific information, even when provided by credible sources such as the Center for Disease Control and Prevention (CDC) (Kricorian et al. 2021). One study, which looked at the psychological characteristics associated with behaviour during Covid-19 pandemic, demonstrates that people who have generally conspiratorial and paranoid beliefs show increased resistance to Covid-19 vaccination (Murphy et al. 2021).

Some studies, which looked at the correlation between gender and vaccine acceptance, found that women are more likely to exhibit increased Covid-19 vaccine hesitancy (Nguyen 2021; Neumann-Böhme et al. 2020; Troiano and Nardi 2021; Schwarzinger et al. 2021; Paul et al. 2021; Shekhar et al. 2021; Coe et al. 2021; Zintel et al. 2022). However, one study conducted in Australia (Seale et al. 2021) found that women were more likely to agree with the statement that “Getting myself vaccinated for Covid-19 would be a good way to protect myself against infection”, which contrasts many other studies and systematic reviews looking at the relationship between gender and Covid-19 vaccine acceptance.

Race is also a frequently addressed aspect when looking at attitudes towards Covid-19 vaccines. In some studies, White and Asian adults had a higher rate of Covid-19 vaccine acceptance (Biswas et al. 2021; Malik et al. 2020) compared to Black adults who had the highest rate of non-intent for Covid-19 vaccination (Nguyen 2021; Shekhar et al. 2021; Troiano and Nardi 2021; Coe et al. 2021). Although in most settings adults of Asian descent were generally more likely to have a positive attitude towards the vaccine, a survey in England found that Asians (alongside Black, mixed, or other non-white backgrounds) were 3 times more likely to reject a vaccine for themselves and their children compared to White respondents (Bell et al. 2020).

Moreover, trust in government institutions is also connected to attitudes towards Covid-19 vaccines. This is based on evidence showing that countries where acceptance exceeded 80% tended to be Asian nations with strong trust in central governments (e.g., China, South Korea and Singapore) (Lazarus et al. 2021). Similarly, government websites were by far the most trusted source of information for respondents from the UAE (Ahamed et al. 2021). Biswas et al. (2021) underscored that mistrust in the authorities was amongst the reasons related to Covid-19 vaccine hesitancy. Similarly, Nguyen (2021) highlighted the lack of trust in the government as a factor undermining vaccination intent. A different study on the psychological profile of the vaccine-resistant individuals discussed how they are more likely to be distrusting of experts and authority figures (Murphy et al. 2021). In Saudi Arabia, higher levels of trust in the healthcare system were associated with higher vaccination willingness (Al-Mohaithef and Padhi 2020). In the USA, randomised controlled experiments showed that public acceptance of Covid-19 vaccines was directly related to the timing of the vaccine (before or after election) and to the statement and stances of elite statesmen, especially the President and the Chief Medical Advisor to the President, Dr. Anthony Fauci (Bokemper et al. 2021).

A recent comparative study from Denmark led by Lindholt et al. (2021) looked at public acceptance of Covid-19 vaccines across eight countries (Denmark, France, Germany, Hungary, Sweden, Italy, United Kingdom, and United States). This survey-based study identified two main reasons behind the willingness to get vaccinated: (1) trust in the national health authorities and scientists, and (2) personal health concerns. The study also found that Denmark was the country where people were most willing to get vaccinated: in this study, the results for Denmark were 82% and the results from the UK were 76%. Although not so different in terms of acceptance rate, the study shows that there is less endorsement of conspiracy beliefs in Denmark than in the UK (Lindholt et al. 2021: 19). Further, the decision of Danish health authorities to withdraw the AstraZeneca vaccine from the vaccination programme led to a reduction in willingness to get vaccinated in Denmark by 11% (Kupferschmidt and Vogel 2021), showing that willingness to get vaccinated was also sensitive to changes in policy and media discourses.

While the above-mentioned studies are helpful for shedding light on current public perceptions of Covid-19 vaccines, the majority are largely based on quantitative analysis (survey studies mainly) and beg further questions about underlying attitudes and factors. This, in turn, demands more fine-grained analysis through qualitative means which is one of the primary contributions of this paper. By conducting semi-structured interviews, we were able to explore in more depth some of the public attitudes and understandings of Covid-19 vaccines, and the values and perceptions underpinning these. In the section that follows, we outline our method and briefly discuss its strengths and limitations before moving on to presenting and analysing our data.

## Methodology

During May and June 2021, we carried out 23 online interviews with members of the UK public, using online meeting platforms such as Microsoft Teams and Zoom. Prior ethical clearance for this study was obtained from the study lead’s (corresponding author's) institution (reference: MRA-20/21-22716). The online interviews were recorded and later transcribed and analysed according to a list of relevant themes. The study participants were recruited using social media. All UK residents above the age of 18 were eligible to participate in the study. Announcements to take part in the study were shared on the authors’ social media handles and within their professional and social circles, including university Facebook and WhatsApp groups. Additionally, other people within these groups were encouraged to share the announcement within their circles. We also posted the announcements in specific Facebook pages and forums that are dedicated to discussing Covid-19 and related vaccines. These include the following Facebook groups: “Covid-19 Research Involvement Group”; “Covid19 vaccine discussion group”; “Against Covid 19 vaccine”; and “We are not against vaccines, but only skeptical about mRNA & DNA vaccines”.

We received 75 responses in total. Those interested in taking part were asked to fill an online form which collected data on three domains: demographics, education, and profession. Additionally, and since our objective was to explore diverse perceptions and attitudes towards Covid-19 vaccines, we also collected data on Covid-19 vaccination status and intention according to whether the participant received the first dose of the vaccine; both doses; neither but hoping/willing to; neither but might to; or neither and refuses to. Purposive sampling was used for selecting participants from the completed online forms to ensure as much diversity as possible across all domains. One group that proved hard to receive responses from were those opposed to vaccination. Despite many attempts to reach out to relevant anti-Covid-19 vaccines groups on social media and relevant forums, we did not manage to receive much interest in participation. The few who did respond and expressed their interest in being interviewed did not show up to the interviews nor responded to follow-up emails. As such, in this category, we only had two participants, making up 8.7% of the total sample. Further limitations of the final sample are discussed below.

The selected participants were contacted to be invited for an online interview and were sent an information sheet on the aims, objectives, and methodology of the study as well as the participants’ role and their right to withdraw from the study at any point. All participants signed a consent form for taking part in the study and having their interviews recorded and used in subsequent publications. In order to preserve the anonymity of participants, pseudonyms have been used and identifying information has been removed.

The interview guide included open-ended questions aimed at exploring participants’ perceptions of Covid-19 vaccines, their understanding of the benefits and risks of the vaccines, the factors and reasons informing their knowledge and opinions regarding the vaccines, and the extent to which they trust the government’s management of the pandemic through vaccines and other mechanisms. Adopting a semi-structured approach to interviewing (Brinkman and Kvale 2015) allowed us to pose all of our interview questions while ensuring that participants’ own reflections and elaborations are sensitively included. This approach also allowed us to go into greater detail about participants’ values and the basis for their attitudes towards Covid-19 vaccines.

As mentioned earlier, the interviews were conducted online, which is a synchronous communication of time but an asynchronous communication of space. Some interviews were recorded in audio format and others in video format depending on the participants’ preferences and what they felt comfortable with. The recorded files were stored on a secure cloud server that is suitable for storing sensitive data in accordance with the Information Security Procedures of the study lead’s institution. At the beginning of the interview, participants were reminded of the study’s aims and objectives and their consent to record the interview was taken again verbally.

Conducting interviews online has several strengths and weaknesses. First, they allow researchers to conduct interviews with participants in an extended geographic area, which was suitable for the study as the aim was to capture perception of people in diverse parts of the UK. Having a wide geographic access also means that researchers can include hard-to-reach populations. Second, they require lower levels of expenditure, time, and logistics, as both the researcher and the participants could be at home or in their offices while the interview was being conducted and would not need any extra equipment or logistics. Third, conducting an interview in a comfortable, familiar environment to the participant gives the participants more comfort and freedom in expressing their opinions. Fourth, as the study was conducted in the midst of the Covid-19 pandemic, the online approach provided the required level of safety for both the interviewers and the participants, minimising the risk of infection and transmission of the virus.

Nonetheless, online interviews have their own limitations. One of their main weaknesses is that the researcher is not able to see the participant when there are no video calls, which makes the researcher lose visual cues and body language, aspects that are useful sources of information when conducting interviews — although one can also argue that the more anonymous aspect of audio calls can provide participants with more ease to freely express themselves and their opinions, leading to richer discussions and more candid accounts (Trier-Bieniek 2012). Second, since there is no standardisation of the place, each participant does the interview in a different setting. In some cases, there could be distractions in the room which prevents the participant from fully engaging with the researcher. Third, since online interviews require the use of online platforms, access to computers/smartphones/tablets and internet literacy are needed by the participants, who are expected to know how to run the platform used to conduct the interview. Technological proficiency may not be something that all participants possess, which poses issues for online interviews. Technological limitations, thus, end up excluding potential participants who do not have access to the needed devices or the necessary digital skills to take part in online interviews. But despite all these noted limitations, recruiting participants and conducting interviews online proved to be an effective way for undertaking our study, especially given the added challenges and constrains generated by the Covid-19 pandemic.

## Data, results, and analysis

In this section, we present and analyse the data emerging from the 23 interviews we conducted between May and June 2021. The table below provides a summary of the vaccination status and demographic information of participants (Table 1). Our analysis is guided by the semi-structured nature of the interviews, focusing mainly on three areas as discussed below: perceptions of Covid-19 vaccines, sources of information on Covid-19 vaccines, and trust towards the government’s response to the pandemic. The contents of the interviews were, first, systematically codified with the help of NVivo, and then codes were grouped under each of the three areas. To make sense of the data, we followed an interpretative narrative approach (Davis and Lohn 2020; McQueen and Zimmerman 2006; Wiles et al. 2005), highlighting the important aspects in the participants’ accounts and placing such narratives in the context of the wider debates and relevant literature, as demonstrated in the next sections. This approach was particularly suited as it allowed a conceptualisation of risk, trust, responsibility, and other major themes as experienced and narrated by the participants themselves. This is while ensuring reference to the research questions and the central objectives of the study.

**Table 1:** Vaccination status and biographic information summary

	Vaccination status and biographic information summary
Vaccine status	Fully vaccinated (4), first dose (8), awaiting first dose (9), refusing the vaccine (2)
Age	18–25 (5), 26–35 (7), 36–45 (4), 46–55 (2), 56–65 (5), 66+ (0)
Gender	Female (12), male (10), non-binary (1)
Ethnicity	White British or White other (14), mixed (2), Black Caribbean (1), South Asian (2), Arab (4)
Level of education	Secondary (4); undergraduate (1), postgraduate (13), PhD (2), not mentioned (3)
Subject area	Health sciences (5), psychology/neuroscience (5), languages and education (3), business (2), engineering (2), physics (1), philosophy (1), theatre (1), high school (1), journalism (1), N/A (1).
Occupation	Students (6), vaccinator (3), theatre/opera (2), researcher (1), training assistant (1), executive assistant (1), software developer (1), local government officer (1), engineer (1), director (1), publishing (1), human rights research (1), new-born screening (1), retired (1), N/A (1)

As can be seen in Table 1, the interview group comprised of 12 females, ten males and one non-binary participant. At the time of interviewing, not all age groups had access to the Covid-19 vaccines. Nevertheless, we managed to select participants with varying vaccination status as we were interested in capturing heterogenous accounts and opinions about Covid-19 vaccines and experiences with the vaccination process. One group we found difficult to engage, as noted before, relates to those who refuse to get vaccinated. Of the 23 participants we interviewed, only two were completely against the vaccine. Their accounts, however, are still important to take into consideration, as they shed light on the rationales and factors influencing some people’s stance against Covid-19 vaccines. In terms of age, we had a relatively balanced distribution across age groups, but no participation from those who are older than 65. This might be due to using online platforms for participants’ recruitment and interviewing. According to a recent report by the Centre for Ageing Better (2021), 32% of those who have never or not recently used the internet in the UK were aged between 50 and 69, and 67% were aged 70 or over. This poses a challenge for researchers who wish to conduct online research with participants from these age groups. As for education, those with postgraduate level of education made up 57% of participants, and 17% had only secondary education.

Around 60% of the participants in our study were White British and from other White backgrounds, whereas there was only one participant from Black Caribbean ethnicity. Notably, this participant was the only one from this ethnicity in the initial pool of 75 respondents who replied to our announcements. The low representation of participants from Black minorities might be due to the underrepresentation of such ethnicities within the online Covid-19 interest groups, discussion forums, and circles from which participants were recruited. This might also have to do with the fact that Black minorities continue to be the group with the highest hesitancy level towards Covid-19 vaccine (Kearney et al. 2021; Asaria et al. 2021; Padamsee et al. 2022), which can also affect their willingness to take part in Covid-19 related research. Farooqi et al. (2022) also note that there is a lack of understanding of the concept of research among BAME groups, which is among the complex factors that constitute a barrier for recruiting BAME participants. While the lack of representation of Black minorities in our study is, admittedly, a major limitation of this research, we have nonetheless managed to include participants from other underrepresented groups including Arab and South Asian participants. Further research will seek to recruit a more representative and ethnically diverse sample.

As mentioned earlier, participants were asked to speak about their perceptions and attitudes towards Covid-19 vaccines, their understanding of the vaccines’ benefits and risks, the sources from which they derive their information and knowledge about the vaccines, and the levels of trust they have vis-à-vis the government’s pandemic response. The following analysis is organised according to these themes.

### Perceptions of COVID-19 vaccines: benefits, risks, and concerns

Existing literature demonstrates how perceptions of vaccines’ efficacy and side-effects can impact attitudes towards the vaccines (Schwarzinger et al. 2021; Neumann-Böhme et al. 2020; Bell et al. 2020). In our study, many participants felt that the benefits of having the vaccine (including lower risk of infection, transmission, and death) outweigh the risks (e.g., allergic reactions or lack of sufficient testing in different contexts). Some participants saw no risk at all. The most common benefit reported by participants is that Covid-19 vaccines can help reduce the number and severity of infections, enabling society to return to normal. As the following quote suggests,

*I think first these people who got vaccinated will not get the virus and this will save some lives. Then after they get vaccinated, they will stop the spreading of the virus […] If this works out, in the end, I think more people will get back to normal, many shops will open, and life will get back to normal.* (Tarek, 26, male, Arab, human rights researcher and journalist, not vaccinated at the time of interview but willing to be)

We now know that this is not entirely accurate, as vaccines can only reduce transmission but not completely prevent it (Prunas et al. 2022; Harris et al. 2021). What is evident, though, is that vaccination helps reduce significantly the likelihood of severe illness and hospitalisation, which is one of the main benefits of Covid-19 vaccines (Tenforde et al. 2021). But Tarek’s perception of the vaccines’ benefits has certainly shaped his attitude and willingness to get vaccinated.

Like Tarek, many participants saw Covid-19 vaccines as a means to return to “pre-pandemic normality.” Some saw the vaccines as a “get out of jail free card” (Spencer, 42, male, White British, opera singer, received first dose of vaccine at the time of interview) in the sense that they can enable the reduction of restrictions (including lockdowns and restrictions on travel and access to events and public spaces). This was more so the sentiment when participants considered the potential introduction of Covid-19 vaccine passports, a mechanism that often comes up in debates on how to manage the pandemic moving forward (Goel and Jones 2021; Ada Lovelace Institute 2021a). For instance, Adam (63, male, White Irish, former teacher, and volunteer Covid-19 vaccinator, had received two doses of vaccine at the time of interview) considered both Covid-19 vaccines and vaccination passports as “important tools in going forward” and traveling safely. However, some participants recognised that not everyone can get vaccinated and not all countries have equitable access to Covid-19 vaccines. On the basis of this, they saw in the potential imposition of vaccination passports, as a requirement for access and travel, a risk of generating new forms of discrimination and inequality.

Some participants were motivated to get the vaccine due to medical reasons, and fear that Covid-19 might exacerbate their pre-existing health conditions. For instance, both Hala (25, female, Arab, student in global health, not vaccinated at the time of interview but might decide to) and Isaac (26, male, mixed ethnic background, student, not vaccinated at the time of interview but hoping to) suffer from asthma and were therefore apprehensive about the ramifications of Covid-19 and the effects it might have on their health. For Isaac, even while sheltering at home, he did not feel safe without a vaccine: “*I'm just at home and not leaving the house, then just the thought of even the delivery driver possibly giving me Covid, when they give me my packages and stuff […] I would rather be vaccinated*” (Isaac, 26, male, mixed ethnic background, student, not vaccinated at the time of interview but hoping to). Hala’s health concerns were compounded by her lack of confidence in the health system’s ability to cope with the pandemic. She expressed her apprehensions in the following way:

*Well, at the beginning it was the whole asthmatic thing because I thought if I got it, it will be a little bit hard to do daily work because of my asthma. It caused me a little bit of anxiety […] The second thing is that I'm flying back to Egypt and the healthcare system is really shattering over there […] I'm too afraid, what if it happens that I get it over there and the healthcare system is crashing, and I might need a ventilator or something like that?* (Hala, 25, female, Arab, student in global health, not vaccinated at the time of interview but might decide to)

Other participants reported that they had no particular stance towards Covid-19 vaccines and just wanted to “get it over with”. This was particularly the case of participants who believed that Covid-19 did not necessarily pose a major risk to their health but acknowledged its potential danger to others, and the inconveniences it has introduced into everyday life. As indicated by the following quote:

*The main motivation would be to […] just be over with it. Take it and just stop worrying about anything in regards to the disease. It's not a real sense of protecting myself or anything […] I've already caught Covid, so I'm not afraid of the virus [… but] when you have all these people suffering around you, of course, I just want to get over with it. (*Amer, 26, male, Arab, engineer, not vaccinated at the time of interview but willing to be)

Underlying some of the participants’ attitudes towards Covid-19 vaccines is also a sense of “social responsibility”, insofar as there was a recognition of the fact that one’s behaviour and actions have implications on others and their health. For Amer, despite considering himself to be “invulnerable” to the coronavirus, he was still willing to get vaccinated to encourage others to do so: “*I would still take the vaccine because when people take the vaccine, it also gives an image to others that you should also take the vaccine. It encourages others to take it as well.*” (Amer, 26, male, Arab, engineer, not vaccinated at the time of interview but willing to be). In a similar vein, Faisal (26, male, Arab, medical doctor, received first dose at the time of interview) described himself as “*a young, healthy person who has a very low risk of catching the virus.*” Yet, being mindful that if he would catch it he might have no symptoms, he decided to take the vaccine to avoid becoming a source of transmission: “*I know that the vaccine itself decreases the transmission of the virus. I decided to take the vaccine because I do not want myself to be part of the transmission of the virus if I get it*” (Faisal, 26, male, Arab, medical doctor, received first dose at the time of interview). The Covid-19 vaccine, in this sense, was perceived as a way of protecting others and keeping them safe: “*To me, it made complete sense to have a vaccine because I was not only protecting myself, but I was protecting anybody else I'd have interactions with.*” (Amy, 64, female, White British, retired teacher and CEO, received both doses of the vaccine).

Relatedly, keeping elderly members of the family safe also featured as a motivation to get vaccinated according to some participants’ accounts: “*I have my 85-year-old grandpa living with us, so obviously, to keep him safe as well and the rest of us. That was my motivation.*” (Darshi, 22, female, South Asian, student in neuroscience and volunteer, received both doses of the vaccine). One can view this willingness to get vaccinated to protect the elderly as a form of “intergenerational solidarity,” a term that started circulating all the more at the beginning of the pandemic. Besides the motivation to get vaccinated in order to protect others, intergenerational solidarity has manifested in various other forms following the outbreak of coronavirus. As Kaye (in Coffey 2020) points out, ‘[e]ver since the pandemic started, we’ve seen mutual aid groups springing out of the soil overnight’ where the young and able-bodied are providing assistance to their infirm and elderly neighbours, be it in terms of picking up and delivering food shopping and emergency parcels, providing medical supplies and arranging foodbank referrals, offering some digitally mediated company to those feeling lonely while in self-isolation, or simply maintaining corporeal distance to show respect towards the vulnerable (see also Ajana 2021; Stjernswärd and Glasdam 2021). At the same time, there is also a sense in which the pandemic has put to test intergeneration solidarity, as young generations are believed to have borne the brunt of the situation more than older generations. According to Hugo Till from the Intergenerational Foundation,

There is a key intergenerational tension at the heart of the pandemic, in that lockdown, social distancing, and other vital public health measures are primarily for the benefit of older people more vulnerable to the virus, while the costs of these measures have been imposed overwhelmingly on young people. The young are two-and-a-half times more likely to work in the worst-affected industries [… and] have suffered most from the casualisation of labour markets […]. But the unequal distribution of the burdens of COVID is not limited to finances. Even the claustrophobia of lockdown was not experienced equally: unsurprisingly, people aged 20–29 have the least average living space per person, and are the least likely to have access to private gardens. (Till 2020)

For Till, such tension is not the product of the pandemic alone but has its roots in what he terms “a backlog of intergenerational injustice,” which saw young people coming out on the bottom precisely because of ‘the accumulative impact of years of intergenerationally unjust public policy in employment, housing, and healthcare’ (Till 2020). Nevertheless, one has to remember also that when it comes to mortality patterns, older adults have been disproportionally affected by the Covid-19 pandemic, at least in the early phase of the pandemic (Levin et al. 2020; O’Driscoll et al. 2020; Kang and Jung 2020). As a statistical study conducted by Yanez and his colleagues demonstrates, of the 178,568 COVID-19 deaths reported in a 6-week sample period from a total population of approximately 2.4 billion people from 16 countries, 153,923 deaths (86.2%) were in individuals aged 65 years or older (Yanez et al. 2020). The vulnerability of the elderly to Covid-19 is something that many participants in our study were acutely aware of, including the youngest participants, and one of the factors behind their willingness to get vaccinated.

Reports have shown that Covid-19 vaccine acceptance can also be affected by public perceptions of vaccine brands and related media discourses. Back in March 2021 and following the suspension of AstraZeneca’s vaccine by several EU countries, an online survey by Eurofound revealed that 34% of respondents were hesitant to take the vaccine compared to 25% before the suspension (Gillespie 2021). Reported risks of blood clots were amongst the reasons behind the increased vaccine hesitancy, as fears over safety and potential side-effects became a major concern. In our study, all participants who received one or both doses of Covid-19 vaccines felt safe doing so. Most had no preference with regard to the vaccine brand and accepted the vaccine they were offered during the rollout. Participants who expressed a preference had a stance against getting the AstraZeneca vaccine for its reported side-effects and blood-clotting risk. Some based their preference on the experiences of others in their network and whether they experienced side-effects or not from a given vaccine brand. For instance:

*Risks are there and certainly, as you'll be aware with the AstraZeneca vaccine, there have been suggestions that there might, I repeat might, be an association with blood clotting*. (Adam, 63, male, White Irish, former teacher, and volunteer Covid-19 vaccinator, had received two doses of vaccine at the time of interview)

*Well, I don't prefer AstraZeneca, because everyone says it got them side-effects. My mom got side-effects, everyone I knew who took AstraZeneca got side-effects, so I'm not preferring AstraZeneca, that's one thing. Most probably, I would prefer something that people I know got. My father got Pfizer, a lot of my friends got Pfizer, my sister got Pfizer. They didn't get really much side-effects. I think I'm basing my preference on the vaccine based on who are the close friends or family members, who took what vaccine and what kind of side-effects that it did give them and this kind of stuff.* (Hala, 25, female, Arab, student in global health, not vaccinated at the time of interview but might decide to be)

One of the two participants who refused to get vaccinated expressed her concerns in the following way:

*Obviously, some of these vaccines they talked about that it could cause clots. I haven't taken the vaccine because I have small blood vessels, I have a stent already in my groin. I've just been told yesterday I might need another one. I'm not prepared to take any vaccine that might mess with my blood, because that's what it does all the clotting and stuff, but everyone is different.* (Pamela, 58, Black Caribbean, new-born hearing screener, not vaccinated and refuses to be)

In addition to blood clots, concerns over fertility was also an issue that came up during the interviews. One female participant stated that “*there is a concern for the younger generation, which was expressed by my friend. She heard I don't know from where, but she works for NHS [… that] if you're 19 or young girl, it [Covid-19 vaccine] might affect fertility. I don't know where that came from, but she was quite persistent that this is true.”* (Yanina, 39, female, White Other, director, not vaccinated at time of interview but might decide to be). Another participant expressed her fertility-related concerns in this way: “*I'm a young, fertile woman. One of my biggest dreams in life is to become a mother and I want to have many children. I'm concerned that, that dream of mine could be compromised if something goes wrong with something that hasn't been tested properly.*” (Jasmine, 33, female, White Other, assistant publisher, not vaccinated at the time of interview but might decide to be).

Although existing research has demonstrated that there is no link between Covid-19 vaccines and infertility (Evans et al. 2021; Schaler and Wingfield 2021), willingness to get vaccinated against Covid-19 continues to be influenced by concerns over the potential long-term impact of the vaccines with regard to fertility and reproductive health. According to the findings of an online survey conducted by Turocy et al. (2021) with fertility patients and those hoping to conceive in the next 6 months (*n* = 284), more than half of the total participants were hesitant to accept Covid-19 vaccines due to fears of ‘birth defects, unknown long-term health effects on children and risk of pregnancy loss’ (Turocy et al. 2021). Another survey study by Diaz et al. (2021) also found that fear of adverse effects on fertility was a major cause of Covid-19 vaccine hesitancy in the United States. In this study, it was found that ‘38% of unvaccinated survey respondents believed that COVID-19 vaccines could negatively impact an individual’s fertility, while approximately one-third remained unsure’ (Diaz et al. 2021: p. 2).

Moreover, concerns regarding the effects of Covid-19 vaccines on the individual’s DNA were also mentioned in our study as well as other attitudinal studies on Covid-19 vaccines. As this statement by participant, Jasmine, demonstrates: “*now we're asking people to willingly change their genetic information with a vaccine that has only been tested for less than a year and just get on board with it and don't ask questions*” (Jasmine, 33, female, White Other, assistant publisher, not vaccinated at the time of interview but might decide to). Such concerns are mainly directed at vaccines which use RNA technologies, as is the case with Pfizer and Moderna Covid-19 vaccines. Unlike conventional vaccines which contain inactivated or attenuated versions of the disease-causing pathogen, RNA vaccines contain instead messenger RNA (mRNA) which provides a set of instructions that direct cells in the body to make proteins specific to the pathogen’s surface. This process enables the immune system to learn to recognise and produce antibodies against the protein with the aim to prevent or fight the disease.

Carmichael and Goodman (2020a) reported that one of the recurring fears often aired on social media was the fear that Covid-19 vaccines would somehow alter the person’s DNA. Referring to various videos that have been widely shared on social media, they pointed out to the claims that Bill Gates has been planning to use a vaccine to manipulate or alter human DNA. A popular video foregrounding such claims is that of Carrie Madej in which she argues that Covid-19 vaccines are ‘designed to make us into genetically modified organisms [… and] hooks us all up to an artificial intelligence interface’ (in Carmichael and Goodman (2020b). Many scientists and media platforms responded to such claims by providing information on how the mRNA vaccines work and why they cannot alter someone’s DNA. As explained by Fox et al. (2021), the human genetic code is made up of a different, but related, molecule to the mRNA vaccine. The two molecules have a different chemistry and are in two different parts of the cell: ‘Our DNA stays in the nucleus. But vaccine mRNA goes straight to the cytoplasm, never entering the nucleus. There are no transporter molecules we know of that carry mRNA into the nucleus’ (Fox et al. 2021).

In our study, many participants thought that a lack of education about vaccines and how they function could potentially put parts of society at risk as a result of vaccine hesitance: “*not everyone is well educated about vaccines […] and how they can help so there’s obviously going to be some resistance from some people to get them which will leave some parts of the society vulnerable”* (Gemma, 26, female, White British, student in psychology, had received first dose of vaccine at the time of interview). Such participants also considered Covid-19 vaccines as a political issue and questioned the role of social media, news platforms, and other information sources in providing objective information regarding the vaccines. This takes us to the next section in which we outline and discuss the sources of information our study participants relied on to learn about the Covid-19 vaccines.

### Sources of information on Covid-19 vaccines

Participants utilised a variety of sources of information to expand their knowledge about Covid-19 vaccines. The selection of trusted sources seemed to correlate with participants’ line of work, education, and lifestyle/environment. For instance, one of the participants is a journalist and he clearly stated that being a journalist made him rely almost totally on written articles from mainstream news platforms. Another participant explained how being a doctor and working as a vaccinator made him rely on medical journals and scientific articles to get information about the vaccines. Participants’ jobs were, as such, an influencing factor as to where they received their information about Covid-19 vaccines and how their views were being shaped:

*I trust them because I'm a journalist. It happens that these are some of the most trusted newspapers. I only depend on the written form of media of these publications. I only read articles. I don't watch TV. I don't watch the BBC on TV. I don't watch their videos or anything on YouTube. I just read their articles online. I trust them. I trust The New York Times because it's the most liberal newspaper in the world and I also trust BBC because it's the governmental official source of media especially when it comes to Covid-19 and all of this fuss about it. I love The Guardians. I believe in them, they're very neutral, and they're very balanced.* (Tarek, 26, male, Arab, human rights researcher and journalist, not vaccinated at the time of interview but willing to be)

*I'm a medical doctor and I read journal reports about the vaccines. At this time, I'm working as a vaccinator, so I have good knowledge about the vaccines, about the side-effects of it […] Usually when I want to go for information, which is based on scientific evidence, I go to the academic like the medical journals to read about the vaccines and the most recent published articles.* (Faisal, 26, male, Arab, medical doctor, had received first dose at the time of interview)

In general, the majority of participants expressed their trust in official sources of information such as the NHS and other government websites, which tallied with the findings of other studies (e.g., Ahamed et al. 2021). Some, like the above quoted participants, also relied on scientific and medical journals, mainstream media platforms, especially the BBC and the Guardian, and scientists or medical staff on social media as valid sources of information regarding Covid-19 vaccines. Two main factors that influenced such choice for the participants were the quality of past reporting on the platforms and how trustworthy their information was, and the extent to which news agencies supported their articles with verifiable resources and expert opinions. As the following statement indicates:

*The key is the sources they're using, because news platforms should always give sound sources to what they publish. A science journalist, for example, will refer to something and they will be able to give often a link to another paper, I can go to the original paper and say, "Yes, they've actually interpreted what's written in that paper very well."* (Adam, 63, male, White Irish, former teacher and volunteer Covid-19 vaccinator, had received two doses of vaccine at the time of interview)

On the other hand, several participants felt that they experienced difficulty in finding objective information regarding Covid-19 vaccines, as they felt mainstream media were biased towards reporting positive outcomes and the benefits of the vaccine “*without actually giving the true picture of the negative effects it's having on some people […:] I am wary of the mainstream media, BBC as well, Sky News, all of it really.”* (Carrie, 49, female, White British, local government officer, not vaccinated at the time of interview and refuses to be). Some participants even expressed their concerns over the bias that researchers and scientific articles may have. They felt that this bias may stem from the fact that even researchers are affected by their own backgrounds, interests and beliefs and that there are other researchers who do not agree on all what was being said. Referring to the State of Science Index survey (2018), Eichengreen et al. (2021: pp. 9–10) argue that individuals feel that ‘scientists, as being self-interested and human, can be unduly influenced by government and corporate agendas’. They also refer to other studies which suggest that “disagreement” among scientists is often interpreted by lay people as evidence that scientific conclusions are biased and based on personal belief rather than data and that the scientists in question are incompetent or untrustworthy. This resonates with some of the statements of our study participants. For instance,

*Well, like most things that are talked about with scientists, there're diverse opinions inside the medical and the scientific community […] The main opinion that's being released inside of our media programs is positive towards vaccines, but there still are doctors and scientists that are releasing their concerns and their scepticism around this actually*. (Jasmine, 33, female, White Other, assistant publisher, not vaccinated at the time of interview but might decide to)

*I know for a fact, for example, that if I want actual reliable information about the vaccine, […] I should check, for example, articles about it like in scientific articles, but even [with] scientific articles, we can’t be sure 100%, because in the end, every article is researched by someone and that someone has his ideologies and then that someone has gotten his sources from somewhere and maybe people did not check where he got his resources from*. (Amer, 26, male, Arab, engineer, not vaccinated at the time of interview but willing to be)

Studies have consistently shown that social media were among the top sources of information regarding Covid-19 vaccines (Chaudhary et al. 2021; Al-Mulla et al. 2021; Belsti et al. 2021). Various Twitter analysis studies were conducted in different countries around the globe (Chen et al. 2021; Shim et al. 2021; Guntuku et al. 2021) showing high levels of engagement of social media platforms in Covid-19 related topics, including the vaccine. In our study, the majority of participants felt that social media had influenced others’ perceptions of Covid-19 vaccines but not theirs. Participants had somewhat contradicting views of social media. While they insisted that they did not think of social media as a reliable source based on which one could form one’s opinion and judgement, they mentioned being involved with, and at times, influenced by social media content in several other answers. For example,

*I’m quite wary of social media […] I am also involved in a lot of Facebook groups. They're not anti-vax. I'm not anti-vax, but they're people who've had the vaccine, and they've just commented on their effects.* (Carrie, 49, female, White British, local government officer, not vaccinated at the time of interview and refuses to be)

Some participants explained that they were not affected by what people said on social media but used the platforms as a way to follow the accounts and pages of their trusted scientists or news agencies: “*I follow nature or science stuff on Facebook […] other people's posts haven't really had an influence”* (Isaac, 26, male, mixed ethnic background, student, not vaccinated at the time of interview but hoping to be); “*It's not like I trust social media. I trust those agencies on social media. On Twitter, you would get some news from The Guardian and then news from The Sun, which is two different newspapers with two different ideologies. It's not like I trust Twitter more than YouTube or anything”* (Amer, 26, male, Arab, engineer, not vaccinated at the time of interview but willing to be). Such participants were able to curate their own “social media ecosystem” by following relevant pages and individuals. Some participants were conscious of their own information “bubble” on social media and the limits of such curated bubbles: *“the danger with social media, not just because of the vaccines, but people are in their bubble and in their information, just in their only echo chamber and […] you only tend to talk to people who agree with you”* (Line, 58, female, White Other, volunteer, had received first dose of vaccine at the time of interview). Many participants were also aware and cautious of misinformation or avoided social media altogether by trying *“not to listen to it [social media] because I think there was so much misinformation out […] I think social media has done more damage than helping”* (Bernard, 39, male, White British, training assistant, had received first dose of vaccine at the time of interview). Or as one participant puts it, “*social media is dangerous. It is something which can damage people's perceptions because unlike peer-reviewed scientific journals, anyone can post anything on social media, be it true, be it false, and be it with the best or worst of intentions.’* (Adam, 63, male, White Irish, former teacher, and volunteer Covid-19 vaccinator, had received two doses of vaccine at the time of interview). Overall, the collective sentiment regarding participants’ awareness and avoidance of misinformation (both pro- and anti-vaccine) can be summarised through the following statement by one of the participants: *“I do read the newspapers and I do listen to the news a lot, but I like to think [that], as a relatively intelligent person, I'm able to discern the difference between fact and opinion”* (Amy, 64, female, White British, retired teacher and CEO, had received both doses of the vaccine)*.*

Moreover, participants gained a considerable amount of their knowledge from interactions with family and friends through talks and conversations, and by witnessing their experiences with being infected with Covid-19 or receiving the vaccine: “*If I got friends telling me that they took Pfizer and they felt nothing and then other friends telling me they took AstraZeneca and they felt really bad for two days, that would make me want to take Pfizer more, to be honest*” (Amer, 26, male, Arab, engineer, not vaccinated at the time of interview but willing to be). The influence of family and friends was greater when they had medical background and were telling information backed by evidence from research and medical journals:

*I live as well with a medical student and I trust his opinion. He's a good friend. Obviously, we had a chat and he has friends who work on Covid vaccine […] he always explains to me why and what and if something is not proven, he's not pushing his own personal opinion [...] He's just honest and he just says it as it is. It's facts.* (Yanina, 39, female, White Other, director, not vaccinated at time of interview but might decide to)

Indeed, participants with medical background or those working in the health field reported being a trusted source of information for others in their surroundings:

*I'm also a part-time student of Spanish. Colleagues on that course know I actually also vaccinate […] and have asked me about what they are worried about in terms of receiving the vaccine and the like. As someone who has actually given the vaccine to many hundreds of people, I'm able to walk them through the process. Again, I can tell them that I've had the vaccine too, as have my family. It's helping reassure and also importantly, helping answer questions that they may have, with an honest and frank interpretation of what the evidence says to us.* (Adam, 63, male, White Irish, former teacher, and volunteer Covid-19 vaccinator, had received two doses of vaccine at the time of interview)

### Trust with regard to the government’s response to Covid-19 pandemic

As mentioned before, trust in government institutions plays a key role in shaping public perceptions of vaccines and willingness to get vaccinated. As Bloom and Chan (in OECD 2021) put it, ‘the most important ingredient in all vaccines is trust.’ In the context of the Covid-19 pandemic, the success of vaccination campaigns is believed to be largely influenced by ‘the extent to which people trust the effectiveness and safety of the vaccines, the competence and reliability of the institutions that deliver them, and the principles that guide government decisions and actions’ (OECD 2021). This chimes with the findings of Jennings et al.’s (2020) and Skinner et al.’s (2020) studies, which demonstrate how the perceived competence of political leaders and scientists has been shaping public trust during the pandemic.

In our study, the majority of participants trust the government with the vaccine rollout. However, the same could not be said about the government’s response during the early stages of the pandemic. Almost all participants expressed discontent regarding how the government managed the pandemic in the beginning. Participants viewed the government’s response as being indecisive and not relying enough on scientific evidence:

*They didn't have a clear plan of what to do, how to manage. First, there was talk about having immunity. They were very late in imposing lockdown, after that, they imposed lockdown and then they opened again and then they wanted to open for Christmas. Then after that, the cases skyrocketed, and then they suddenly abruptly decided to cancel Christmas. There's a feeling of indecisiveness in the government.* (Amer, 26, male, Arab, engineer, not vaccinated at the time of interview but willing to be)

*Originally, they said that they were looking to have herd immunity, which at first sounded quite scary, but actually, […] I thought [it was] a very good way to build your immune system. That's what we do with flu and lots of other things. I felt that the government backtracked and then I feel that they scaremongered a lot of people.* (Carrie, 49, female, White British, local government officer, not vaccinated at the time of interview and refuses to be)

Some participants also thought that the government politicised the pandemic by treating it as a “political game” to advance their agenda and interests. As this statement illustrates: “*I think it was more influenced as a political game. I think that is unfortunate […] everything that is done is done as a political gain of some sort. It's not done [like:] "This is the right thing to do let's just do it. It doesn't matter if I'll lose my election because of this, but I'll save people".”* (Yanina, 39, female, White Other, director, not vaccinated at time of interview but might decide to).

With regard to the vaccination programme and how the government has managed the pandemic since the rollout of the Covid-19 vaccines, there was an overall agreement that the programme was a success, even among the participants who had scepticism towards the vaccine itself:

*I think that the vaccine programme […] has been done really well […] I do think of most of the things that Boris Johnson has been involved in with this pandemic, this is probably one of his successes; the vaccine*. (Jasmine, 33, female, White Other, assistant publisher, not vaccinated at the time of interview but might decide to)

*If you want to talk about the vaccine program, I can say that they are managing well. They are really doing a great job. At that moment, more than 70% of adults who are eligible to take the vaccine in the UK get their first jab which is really great. Now we started to see that the effect of the vaccine started to show up, as the lockdown eased, the stores reopened again, people start to meet again. The way that the government managed the vaccination program is done very well.* (Faisal, 26, male, Arab, medical doctor, had received first dose at the time of interview)

However, some participants felt that the credit for the success of the Covid-19 vaccine rollout should be given to scientists and healthcare workers, such as the NHS or the Royal Voluntary Service: “*Do I trust the government? No. Do I trust what they've done with the vaccine? Yes, but it's not been them really it's been the scientists and then the health service.”* (Arthur, 56, male, White British, stage manager, had received first dose of vaccine at the time of interview).

When asked about their views on vaccine passports as a mechanism of managing the pandemic, some participants expressed mistrust in the government’s ability to securely store data and maintain privacy. They feared potential data leak or misuse. This concern was not only vis-à-vis vaccination passports but also other apps that have been deployed to trace the spread of coronavirus: “*I wish the government will get a handle on the leakiness of the information that comes from the apps. The other day was discovered that the app was leaking information about people's status and so it is really, really poor. I work for a tech giant and it is really easy to design an app that keeps the information secure. It's pretty poor that we can't do that*” (Joanna, 48, female, White Other, executive assistant, had received first dose of vaccine at the time of interview). Other studies exploring public perceptions of Covid-19 vaccination passports also demonstrate how members of the public are wary of the adoption of these passports as a means of managing the pandemic (Ada Lovelace Institute 2021b). In addition to privacy and data protection concerns, there are also concerns about the issue of “function creep”, which in this context refers to the potential use of these digital passports and the personal information they contain for purposes beyond those initially planned and declared (Ada Lovelace Institute 2021b).

The level of trust with regard to actual governmental responses to Covid-19 pandemic seemed to also influence participants’ views on managing the pandemic going forward. Almost all participants agreed on continuing the vaccination programmes until a level of herd immunity, ranging from 70% to 90%, is reached to enable return to some sort of normality. However, their views differed when it came to other ways of managing the pandemic, including the deployment of vaccination passports or the maintenance of restriction measures, as some feared these would impinge on human rights. Importantly, some participants explained that the rollout of Covid-19 vaccines should be done on a global level. They argued that vaccines should be donated to lesser fortunate countries to ensure global safety, stressing that rich countries have the moral responsibility to donate vaccines to poorer countries: “*Actually, for the wealthy countries, they should take part in providing all the support, the financial support for other countries to get the vaccine either in terms of the vaccine itself or facilitating the administration of it. For me, this will be the only thing that could be done to end the pandemic because it affect everyone in the world, and everyone in the world could be the source of infection to other people because we are open to each other”* (Faisal, 26, male, Arab, medical doctor, had received first dose at the time of interview). As such, global vaccine solidarity, so to say, is seen not only as a moral imperative but also as a pragmatic way of reaching herd immunity globally and, ultimately, bringing the pandemic to an end.

## Discussion

The findings of this study contribute to existing attitudinal research on Covid-19 vaccines. Our focus has been on examining the public perceptions of risks, benefits, and concerns relating to the rollout of Covid-19 vaccines as well as contributing factors to such perceptions, including the level of trust in government response to the pandemic and the sources from which participants derive information about the vaccines. The narratives obtained from participants highlight multifaceted, and at times contradictory, attitudes towards Covid-19 vaccines and the overall response to the pandemic. While the majority of participants saw multiple benefits in the vaccination programmes against Covid-19, some were also wary of the perceived risks relating to side-effects, fertility, DNA, and the “speed” with which the vaccines have been developed. This resonates with the findings of other research (Neumann-Böhme et al. 2020; Bell et al. 2020; Troiano and Nardi 2021; Biswas et al. 2021; Nguyen 2021) which links vaccine acceptance rates to perceptions of the vaccines’ safety, efficacy, and risks. At the same time, our findings provide some nuance and context to existing surveys by identifying the social and mediated factors influencing public perceptions and attitudes. Some interesting themes emerged in this regard.

First, some participants did not perceive Covid-19 as representing much risk to their health, yet felt a sense of responsibility towards others which compelled them to get vaccinated. In this sense, vaccine attitudes are not reducible to perceptions of the vaccines themselves, but tend to be informed also by perceptions of one’s *own* health and level of susceptibility to infection as well as perceptions of the risks and effects of the virus itself on *others*. This adds a communal dimension to risk perception at the individual level, playing an important role in vaccination intentions. This also links to the notion of solidarity we mentioned earlier, aspects of which have been highlighted in participants’ narratives. Other studies have also sought to articulate the link between solidarity and vaccination intention. For instance, Wakefield and Khauser (2021) argue, following the findings of their survey, that community identification and the sense of duty to others predict willingness to receive a Covid-19 vaccine. Another survey study conducted by Patzina and Dietrich (2022) on German adolescents measured the extent to which participants felt that getting vaccinated against Covid-19 constitutes an expression of societal solidarity. The findings indicate that ‘solidarity beliefs explain almost 40% of the variation in vaccination intentions against COVID-19 among adolescents independent of general risk preferences, personality, interpersonal trust, and time preferences’ (Patzina and Dietrich 2022: p. 8). Therefore, the authors conclude that ‘an individual’s decision to be vaccinated is perceived to be an act done for the common good in contrast to purely a self-interested action’ (Patzina and Dietrich 2022: p. 8). They see this as an area of conflict in individual decision-making that needs further research and investigation to test the effects of self-interest and common good orientations in vaccine uptake.

Uncertainty was also a recurring theme in participants’ narratives. First, some participants took issue with the scientific uncertainty surrounding Covid-19 vaccines, particularly with regard to whether the vaccines have been sufficiently tested, whether they are effective enough in terms of transmission and protection, and whether they had any long-term effects that would only become known with time. Such concerns were also evident in the findings of a qualitative study conducted by Williams (2022) on attitudes towards Covid-19 vaccines in children whereby parents expressed the need for more certainty and evidence of testing and safety to feel more confident. Secondly, uncertainty also relates to the ways in which the benefits and risks of Covid-19 vaccines were *communicated* to the public and confusion caused by changing messages and policies, which was interpreted as indecisiveness by some of our study participants, as discussed before. While recognising the challenge of ‘discussing newly licensed vaccines for an emerging and uncertain disease’ (Kelp et al. 2022), Kelp and her co-authors argue that the way scientific uncertainty is communicated to the public has an impact on public attitudes. Through a survey study they conducted with college students, they examined the extent to which “uncertainty communication” had an effect on risk perceptions, trust in science and government, and behavioural decision-making, including vaccine uptake. One of their conclusions is that ‘individuals who read information with low uncertainty ranked the safety and efficacy of the COVID-19 vaccine higher’ (Kelp et al. 2022: 233). However, information with “low uncertainty” might not always be a “true” reflection of the state of what is being communicated, in this case the safety and efficacy of Covid-19 vaccines. This raises the question of ‘ethics involved in the need to disclose the limitations and uncertainty of science to patients’ (Kelp et al. 2022: 234) and the possible trade-off between ‘short-term and long-term understanding and trust’ (Kelp et al. 2022: 234).

Trust is, indeed, another important theme pertaining to this discussion. In our study, participants accorded varying degrees of trust to the different actors involved. While showing a relatively high level of trust in scientists and healthcare institutions, the majority of participants expressed dissatisfaction and, at times, a sense of mistrust towards the government, taking issue with the way it handled the pandemic especially at the beginning. These sentiments were not only due to the perceived indecisiveness of the governments or how it communicated uncertainty around Covid-19 vaccines, but also in terms of underplaying the risks of Covid-19 and seemingly embracing the herd immunity approach at the beginning of the pandemic. As one participant puts it: “*Remember Johnson was also on record as saying in March that his view was maybe it would be best to let the virus rip through the community. In other words, to cull the weak and the sick*.” (Adam, 63, male, White Irish, former teacher, and volunteer Covid-19 vaccinator, had received two doses of vaccine at the time of interview). This point resonates with Williams’ (2022) study participants for whom the lack of trust in government was based on what they perceived as ‘past failures.’ In our findings, the government’s “delayed” response to the pandemic was also perceived as a form of failure, impacting participants’ trust. Interestingly though, and as mentioned in the preceding section, this did not affect much the perceptions towards the Covid-19 vaccination programme itself, as even those who were uncertain about the vaccine thought the programme was a success. As such, and when studying the relationship between trust and vaccine acceptance or hesitancy, it is important to attend to such variations and nuances. This includes the other technologies at the periphery of vaccines, e.g., vaccine passports and immunity certificates. Perceptions and trust with regard to these technologies tend to also affect attitudes towards the vaccines, something that came through the narratives of some of our study participants. Evidence from a large-scale national survey in the UK, conducted by de Figueiredo et al. (2021), suggests that vaccine passports may result in a *lower* inclination to accept Covid-19 vaccines. This decrease is larger if passports were required for domestic use rather than for international travel, according to the study. Such findings have implications for policy on vaccine certification given the potential of such techniques to lower vaccination inclination in those who are wary of pandemic surveillance technologies (even if not opposed to the vaccine itself) and in socio-demographic groups with less confidence in Covid-19 vaccines.

Trust also featured in discussions around the sources of information on Covid-19 and vaccines. Our study participants trusted information that is largely coming from official sources such as government websites and the NHS. This contradicts other studies suggesting that there has been a decline in trust towards government institutions and healthcare providers as a source of information on Covid-19 (Boyle et al. 2021; Ali et al. 2020). Some participants also described traditional media outlets, such as newspapers and national television as another highly trusted source of information. Other studies (Boyle et al. 2021; Purvis et al. 2021; Latkin et al. 2021), however, suggest that news media are seen as less trustworthy when compared to other sources of information about Covid-19 vaccines. Medical and scientific journals were also considered by some participants as a trustworthy and reliable source of information which links to trust in science and scientists (Sturgis et al. 2021; Latkin et al. 2021; Purvis et al. 2021). Some participants also reported that friends and family members, especially those who already received the vaccine and those who work in the medical sector, were a valuable and trusted source of information on Covid-19 vaccines. Williams (2022) refers to ‘local social norms’, i.e., ‘the views and beliefs of immediate network of family, friends, and close others’ (Williams 2022: 118), as being influential on participants’ views on the vaccines. Similarly, a significant number of respondents in Purvis et al.’s (2021) study described personal relationships, family, and friends as their trusted sources of information about Covid-19 vaccines.

Contradictive views emerged vis-à-vis social media. While there was a general sense of wariness in our study participants’ accounts regarding the trustworthiness of social media as a source of information, some participants also mentioned being involved with social media groups and, sometimes, being influenced by the content of their posts. Others used social media to follow the accounts and posts of those they deemed as trusted scientists and journalists. Overall, participants believed that social media was a major source of misinformation which could have a negative impact on the vaccination programmes. These views and concerns resonate with various academic studies that link vaccine hesitancy to the spread of misinformation and conspiracy beliefs on social media (Featherstone et al. 2019; Jennings et al. 2021). The relatively unregulated nature of social media sources makes it hard for some users to discern what is factual and what is not, which can impact perceptions and attitudes with regard to Covid-19 vaccines (Chadwick et al. 2021; Muric et al. 2021). Added to this is the issue of “information bubble” or “filter bubble” which leaves users contained within echo chambers and exposed to content that has been curated and tailored according to their previous searches, views, and digital profile. As Hussein et al. (2020) explain in relation to YouTube videos, ‘once a user develops a watch history, these attributes do affect the extent of misinformation recommended to them […] watching videos that promote misinformation leads to more misinformative video recommendations.’ It is for such reasons that Jennings et al. (2021) call for more action from governments, health officials, and social media companies to help users understand their own risks and fill existing knowledge gaps.

## Conclusions

In this paper, we built on existing literature and drew on 23 in-depth interviews with members of the UK public to examine perceptions and attitudes towards Covid-19 vaccines. Our findings indicate that participants generally felt that the benefits of having the vaccine outweigh the risks and that Covid-19 vaccines are a crucial mechanism for enabling society to return to normal. Vaccine acceptance was, for some, strongly linked to a sense of social responsibility and the duty to protect others. However, some participants expressed concerns towards the side-effects of Covid-19 vaccines and their perceived potential impact on fertility and DNA makeup. Participants used various sources of information to learn about Covid-19 vaccines and understand their function, benefits, and risks. These sources seemed to correlate with participants’ line of work, education, and lifestyle/social environment. News outlets, scientific articles, and social media were all major information sources for participants. Some were wary, however, of the limitations of such sources with regard to accuracy and neutrality as well as of the issue of “information bubbles” which delimits what users have access to and with whom they interact. In terms of trust in the government’s handling of the pandemic, there was an overall agreement that the vaccine rollout was a success. However, the majority of participants criticised the government’s response during the early stages of the pandemic, given the government’s perceived indecisiveness, laissez-faire approach, and embracing of herd immunity before the development of a viable vaccine during that early period of the pandemic. These findings are useful for understanding how members of the public make sense of the Covid-19 vaccines and the factors influencing their attitudes towards such artefacts of pandemic governance. To support an effective immunisation campaign that is capable of bringing the pandemic to an end, governments need to understand public concerns, garner trust, and devise adequate strategies for engaging the public and building more resilient societies.

## Data Availability

Not applicable
